# Enhanced production of poly(3-hydroxybutyrate) in recombinant *Escherichia coli* and EDTA–microwave-assisted cell lysis for polymer recovery

**DOI:** 10.1186/s13568-018-0672-6

**Published:** 2018-09-04

**Authors:** Aneesh Balakrishna Pillai, Arjun Jaya Kumar, Harikrishnan Kumarapillai

**Affiliations:** Environmental Biology Laboratory, Rajiv Gandhi Centre for Biotechnology (RGCB) Poojappura, Thycaud P. O., Thiruvananthapuram, Kerala 695014 India

**Keywords:** Poly(3-hydroxybutyrate), Recombinant *E. coli*, EDTA, Microwave, Cell lysis, Polymer extraction

## Abstract

Poly(3-hydroxybutyrate) (PHB) is a bacterial polymer of great commercial importance due to its properties similar to polypropylene. With an aim to develop a recombinant system for economical polymer production, PHB biosynthesis genes from *Bacillus aryabhattai* PHB10 were cloned in *E. coli.* The recombinant cells accumulated a maximum level of 6.22 g/L biopolymer utilizing glycerol in shake flasks. The extracted polymer was confirmed as PHB by GC–MS and NMR analyses. The polymer showed melting point at 171 °C, thermal stability in a temperature range of 0–140 °C and no weight loss up to 200 °C. PHB extracted from sodium hypochlorite lysed cells had average molecular weight of 143.108 kDa, polydispersity index (PDI) 1.81, tensile strength of 14.2 MPa and an elongation at break of 7.65%. This is the first report on high level polymer accumulation in recombinant *E. coli* solely expressing PHB biosynthesis genes from a *Bacillus* sp. As an alternative to sodium hypochlorite cell lysis mediated polymer extraction, the effect of combined treatment with ethylenediaminetetraacetic acid and microwave was studied which attained 93.75% yield. The polymer recovered through this method was 97.21% pure, showed 2.9-fold improvement in molecular weight and better PDI. The procedure is simple, with minimum polymer damage and more eco-friendly than the sodium hypochlorite lysis method.

## Introduction

Polyhydroxyalkanoates (PHAs) are a group of polyesters accumulated in microorganisms as intracellular granules in response to unbalanced growth conditions (Rehm 2003). In bacteria, PHAs act as a reserve for carbon and energy and are utilized when external supply of carbon is limited (Gao et al. 2001). Their molecular weight and monomer composition varies depending on the microorganism and the growth conditions, which reflect in their physical properties (Urtuvia et al. 2014). Based on chain length of PHA monomers, they are grouped into three: short-chain-length (SCL, 3–5 carbon atoms), medium-chain-length (MCL, 6–14 carbon atoms) and SCL–MCL PHA copolymers (containing SCL as well as MCL monomers) (Phithakrotchanakoon et al. 2013; Pillai and Kumarapillai 2017).

Bacterial PHAs are regarded as a green-substitute for synthetic plastics due to their complete biodegradability in environment, possibility to produce from renewable resources, and plastic-like properties. But their wide spread application as commodity plastics is hampered mainly by high cost of production and extraction from bacterial biomass (Muhammadi et al. 2015). To circumvent this, several alternative approaches including the use of cheap substrates and hyper producing bacterial strains are being tested (Khanna and Srivastava 2005; Madison and Huisman 1999). PHA production from recombinant *E. coli* is a proven strategy for improvement of polymer yield and easier polymer recovery (Lee et al. 1994; Lee and Chang 1995; Lee 1997). *E. coli* is an ideal host for PHA production owing to several advantages over wild‐type PHA producers such as their faster growth rate and ability to grow at high cell densities utilizing easily available cheap carbon sources (Lee 1996; Madison and Huisman 1999). In addition, the fragility of *E. coli* cells facilitates easy recovery of the polymer and as they do not produce any PHA depolymerase enzymes, the accumulated PHAs are not utilized (Choi and Lee 1999; Li et al. 2007).

Recovery of polymer from bacterial cell mass is the major factor contributing more than 50% of the PHA production cost (Samori et al. 2015a, b). An ideal polymer extraction process should be simple, inexpensive, environmental friendly, yielding polymer of high purity and of higher recovery rate (Heinrich et al. 2012). Several studies have been reported for polymer recovery using organic solvents, chemicals, enzymes, mechanical disruption, etc. (Ramsay et al. 1994; Berger et al. 1989; Kapritchkoff et al. 2006; Pötter and Steinbüchel 2005; Tamer et al. 1998; Heinrich et al. 2012). But at industrial scale, most of these processes are found to be either affecting the quality of the extracted polymer or increasing the overall production cost. The widely used methodology, utilizing sodium hypochlorite solution for cell lysis and polymer release, cause severe degradation of polymer chains resulting a considerable reduction in molecular weight of the extracted PHA (Berger et al. 1989). This demands development of new strategies for the extraction of high quality PHAs from bacterial cell mass with minimum environmental pollution and maximum polymer recovery.

PHB is a SCL-PHA with physical properties similar to that of polypropylene (Griffin 1994) and has tremendous application potential in various fields (Sharma and Ray 1995). *B. aryabhattai* PHB10 is an efficient PHB accumulating strain described in our previous studies (Pillai et al. 2017a, b). In this study we engineered a recombinant *E. coli* accumulating PHB using biosynthesis genes from *B. aryabhattai* PHB10 and evaluated a microwave–EDTA treatment method for cell lysis in perspective of easier and more eco-friendly polymer extraction.

## Materials and methods

### Bacterial strains, plasmids and primers

The bacterial strains, plasmids and oligonucleotide primers used in this study are listed in Table 1.

**Table 1:** Bacterial strains, plasmids and primers used in this study

Strain/plasmid/primer	Relevant characteristics	Reference/source
Bacterial strains		
*B. aryabhattai* PHB10	PHB accumulating environmental isolate	Pillai et al. (2017a), MTCC Accession no. 12561
*E. coli* JM109	*end*A1, *gln*V44, *rec*A1, *gyr*A96, *thi, hsd*R17 (r_k_^−^, m_k_^+^), *rel*A1, *sup*E44, Δ(*lac*-*pro*AB), [F′ *tra*D36, *pro*AB, *laq*I^q^ZΔM15]	Promega Corporation
Plasmids		
pUC18	Cloning vector; Amp^R^	Thermo Fisher Scientific
pGEM-T easy	T/A cloning vector; Amp^R^	Promega Corporation
pTZ57R/T	T/A cloning vector; Amp^R^	Thermo Fisher Scientific
pHB4485	pUC18 carrying 4.48 kb PHA gene cluster from *B. aryabhattai* PHB10	This work
pHB5803	pUC18 carrying 4.48 kb PHA gene cluster and *phaA* from *B. aryabhattai* PHB10	This work
Primers		
PHA4485F	5′-GTTACCCCAAATTCTTGAGC-3′	This work
PHA4485R	5′-CAGGAGTCTTCGCCTTGC-3′	This work
PHA1278F	5′-GAAAGGAAATTGAGCAAGCG-3′	This work
PHA1278R	5′-TGCTCCAATAACCATAACTG-3′	This work
T7 promoter primer	5′-TAATACGACTCACTATAGGG-3′	Promega Corporation
SP6 promoter primer	5′-TATTTAGGTGACACTATAG-3′	Promega Corporation
M13/pUC sequencing primer	5′-GCCAGGGTTTTCCCAGTCACGA-3′	Thermo Fisher Scientific
M13/pUC reverse sequencing primer	5′-GAGCGGATAACAATTTCACACAGG-3′	Thermo Fisher Scientific

### DNA manipulations

DNA isolation, restriction enzyme digestion, DNA ligation and agarose gel electrophoresis were performed by standard protocols (Sambrook and Russell 2001) and following the manufacturer’s instructions. Restriction enzymes were purchased from Thermo Fisher Scientific (Massachusetts, USA). DNA purification was carried out with Illustra GFX PCR DNA and Gel Band Purification Kit (GE Healthcare, Illinois, USA) and DNA transformation was carried out according to Hanahan (1985).

### Construction of recombinant vector system

A 4.48 kb DNA fragment, *phaPQRBC* coding for PHA gene cluster and 1.2 kb fragment of acetyl-CoA C-acetyltransferase (*phaA*) were amplified with the primer sets PHA4485F–PHA4485R and PHA1278F–PHA1278R respectively, using genomic DNA of *B. aryabhattai* PHB10 as template. The *phaPQRBC* was ligated with pGEM-T Easy vector (Promega Corporation, Wisconsin, USA) and the *phaA* was ligated with pTZ57R/T cloning vector (Thermo Fisher Scientific). The ligation mixtures were independently transformed into *E. coli* JM109 chemical competent cells. From the resulting plasmids, the multiple cloning sites harbouring the inserts were amplified using vector specific primers. The PCR product harbouring *phaPQRBC* insert was digested with *Eco*RI and ligated to pUC18 linearized with *Eco*RI. The ligation mixture was transformed into chemically competent *E. coli* JM109 cells to obtain the plasmid pHB4485. *phaA* fragment was inserted into pHB4485 by *Xba*I–*Bam*HI double digestion and subsequent ligation. The resulting plasmid pHB5803 confers ampicillin resistance and PHA biosynthesis property to the *E. coli* cells.

The sequences of *phaPQRBC* and acetyl-CoA C-acetyltransferase (*phaA*) are available at NCBI GenBank under accession numbers NOXE01000006.1 and NOXE01000003.1.

### Culture conditions

Recombinant *E. coli* strains were grown at 37 °C in Luria–Bertani (LB) medium supplemented with 100 µg/mL ampicillin. The medium was solidified with 15 g/L agar, when required. For blue–white screening, 4-chloro-3-indolyl-β-d-galactopyranoside (X-gal) and isopropyl-β-d-thiogalactopyranoside (IPTG) were added to the culture medium at final concentrations of 50 µg/mL and 0.5 mM respectively. Shake-flask experiments for polymer production in recombinant *E. coli* were performed with M9 minimal medium containing 10 g/L glycerol as carbon source at 37 °C with agitation rate of 250 rpm. The medium was supplemented with 0.67 g/L yeast extract, 1.2 g/L peptone and 0.1 g/L ampicillin. *B. aryabhattai* PHB10 was cultured in basal medium supplemented with 20 g/L glucose for polymer accumulation at 30 °C and 180 rpm (Aneesh et al. 2016). After 48 h fermentation, the cells were harvested by centrifugation at 5000 rpm, lyophilized and the cell dry mass (CDM) was calculated. The fermentation studies were conducted in triplicate and the mean values were taken. The polymer was extracted from the cells according to Shi et al. (1997) and quantified spectrophotometrically after converting PHB to crotonic acid by sulphuric acid treatment (Law and Slepecky 1961).

### Visualization of polymer accumulated cells

#### Sudan Black B staining

The staining was carried out according to Burdon (1946). A thin smear was prepared on a glass slide, heat fixed and stained with Sudan Black B solution (0.05% in ethanol) for 10 min. It was followed by destaining in xylene for a few seconds and counter staining with 0.5% safranin for 5 min. The smear was observed under 100× oil immersion objective lens of light microscope Nikon YS100 (Nikon Corporation, Tokyo, Japan).

#### Nile Red staining

Nile Red staining was performed as described by Jendrossek et al. (2007) with modifications by Aneesh et al. (2016). The cells were imaged on a Nikon A1R-Si laser scanning confocal spectral microscope with 50× magnification (Nikon Corporation, Tokyo, Japan) excited at 561 nm.

#### Scanning electron microscopy (SEM)

SEM analysis was conducted by following the protocol described by Soo-Hwan et al. (2011) with slight modifications by Pillai et al. (2017a). After the fixation and drying, 5 μL of the cell suspension was sputter coated with gold and analyzed in a Scanning Electron Microscope JEOL Model JSM-6390LV (JEOL USA, Inc., Massachusetts, USA).

#### Transmission electron microscopy (TEM)

TEM analysis was performed according to Akai et al. (2011) with slight modification. Polymer accumulated bacterial cells were harvested, washed in phosphate buffered saline and fixed in 4% paraformaldehyde and 2% glutaraldehyde in 200 mM sodium phosphate buffer (pH 7.3) followed by a post fixation in 1% osmium tetroxide in 50 mM sodium phosphate buffer. The samples were dehydrated by washing with increasing concentrations of methanol and embedded in Epon812 resin. Ultra-thin sections were taken and stained with uranyl acetate and lead citrate solutions. The sections were imaged with a TECNAI 200 kV transmission electron microscope (Fei, Electron Optics, Oregon, USA).

### EDTA–microwave assisted cell lysis and polymer extraction

The cell lysis studies were carried out in polymer accumulated recombinant *E. coli* cells. The experiment was also conducted in PHB accumulated *B. aryabhattai* PHB10 cells, as a representative of Gram positive bacteria. Cell mass harvested from 250 mL bacterial culture was suspended in 10 mM EDTA solution and kept for one h at room temperature with intermittent shaking. The cell suspensions were then kept individually at the centre of a 2450 MHz microwave oven (Whirlpool MagiCook 20L Classic-S) and exposed to microwave at maximum power (700 W) for 10 min. Microwaving was stopped at regular intervals to prevent frothing of the suspension. After the treatment, cells were pelleted, washed with distilled water and ethanol. The pellet was suspended in boiling chloroform and filtered through glass wool. The filtrate was poured into glass Petri plates and the polymer films obtained were kept open for 1 week in room temperature for complete evaporation of the solvent. Polymer extracted with chloroform from bacterial cells treated with sodium hypochlorite (Shi et al. 1997; Aneesh et al. 2016) was kept as control. The purity (%) of the extracted polymer was calculated as:

$$purity \;\left( \% \right) = \frac{m}{M} \times 100$$

where ‘*m*’ is the mass of polymer as quantified spectrophotometrically and ‘*M*’ is the total mass of sample used for the analysis, as described by Bhattacharyya et al. (2012).

The polymer yield (%) was calculated as:

$$yield\; \left( \% \right) = \frac{m \times p}{CDM \times PHB} \times 100$$

where ‘*m*’ is the mass of the polymer film obtained after extraction, ‘*p*’ is the purity percentage, ‘*CDM*’ is the cell dry mass taken and ‘*PHB*’ is the percentage polymer content (Fiorese et al. 2009).

### Characterization of extracted polymer

#### Gas chromatography–mass spectrometry (GC–MS) analysis

Samples for GC analysis were prepared by methanolysis of the extracted polymer (Juengert et al. 2018). A mixture of 1 mL chloroform and 1 mL acidified methanol (15% v/v H_2_SO_4_) was added to 10 mg of polymer sample and incubated in an oil bath at 100 °C for 2 h. Phase separation was achieved by adding 1 mL deionised water and 1 mL chloroform containing an internal standard (0.2% v/v methyl benzoate) to the mixture. The bottom organic phase was collected, dehydrated with anhydrous Na_2_SO_4_ and 1 μL of the sample was injected directly into the gas chromatograph Shimadzu GC–MS QP2010S fitted with a Rxi-5Sil MS (30 m × 0.25 mm × 0.25 μm) capillary column. The carrier gas (Helium) flow rate was set at 1 mL/min and the injection temperature was 280 °C. The initial column temperature of 90 °C was maintained for 3 min, then increased to 190 °C at the rate of 7 °C/min, held for 5 min and then finally increased to 270 °C at the rate of 8 °C/min, and held for 5 min. After a solvent cut time of 3.6 min, mass spectra were recorded under scan mode in the range of 50–500 m/z. The peaks were identified by comparing to the mass spectral libraries (NIST 17 and Wiley).

#### Nuclear magnetic resonance (NMR) spectroscopy

Proton NMR (^1^H NMR) spectra were recorded in model BrukerAvance^II^ 500 NMR spectrometer (Bruker Corporation, Massachusetts, USA) at 500 MHz and magnetic field strength of 11.7 T. ^13^C NMR analysis was carried out in BrukerAvance^III^ 400 NMR spectrometer at 400 MHz and 9.4 T. The polymer samples suspended in high purity deuterochloroform (CDCl_3_) were used for the tests (Salgaonkar et al. 2013). The spectra were compared with standard PHB (Sigma Aldrich, Missouri, USA).

#### Differential scanning calorimetry (DSC)

2–5 mg of polymer sample was taken for DSC analysis in PerkinElmer DSC6000-Pyris Series instrument (PerkinElmer, Inc., Massachusetts, USA) under flowing nitrogen atmosphere at a heating rate of 10 °C per min (Gunaratne et al. 2004).

#### Thermo gravimetric analysis (TGA)

TGA was carried out over a temperature range of 28–600 °C at a heating rate of 10 °C/min in SDT Q600 V8.3 Build 101 thermal analyzer instrument (TA Instruments, Inc., Delaware, USA) (Salgaonkar et al. 2013).

#### Gel permeation chromatography (GPC)

The analysis conducted in a Waters HPLC system (Waters Corporation, Massachusetts, USA) with chloroform as eluent as described by Su (2013) and Qi and Rehm (2001). Polystyrene standards of molecular weight 1,865,000, 34,300 and 685 Da were used for relative calibration.

#### Tensile properties

Tensile characteristics of the polymer were tested in a universal testing machine (UTM) (Make: Tinius Olsen, Model: 50ST) (Tinius Olsen TMC, Pennsylvania, USA) under room temperature with a 50 kN load cell at fixed cross-head speed of 50 mm/min. Three independent tests were carried out following the ASTM (American Society for Testing and Materials) standard (D882-12) procedure and average values were taken.

## Results

### Expression of PHA genes in *E. coli* and visualization of polymer accumulated cells

**Fig. 1:**
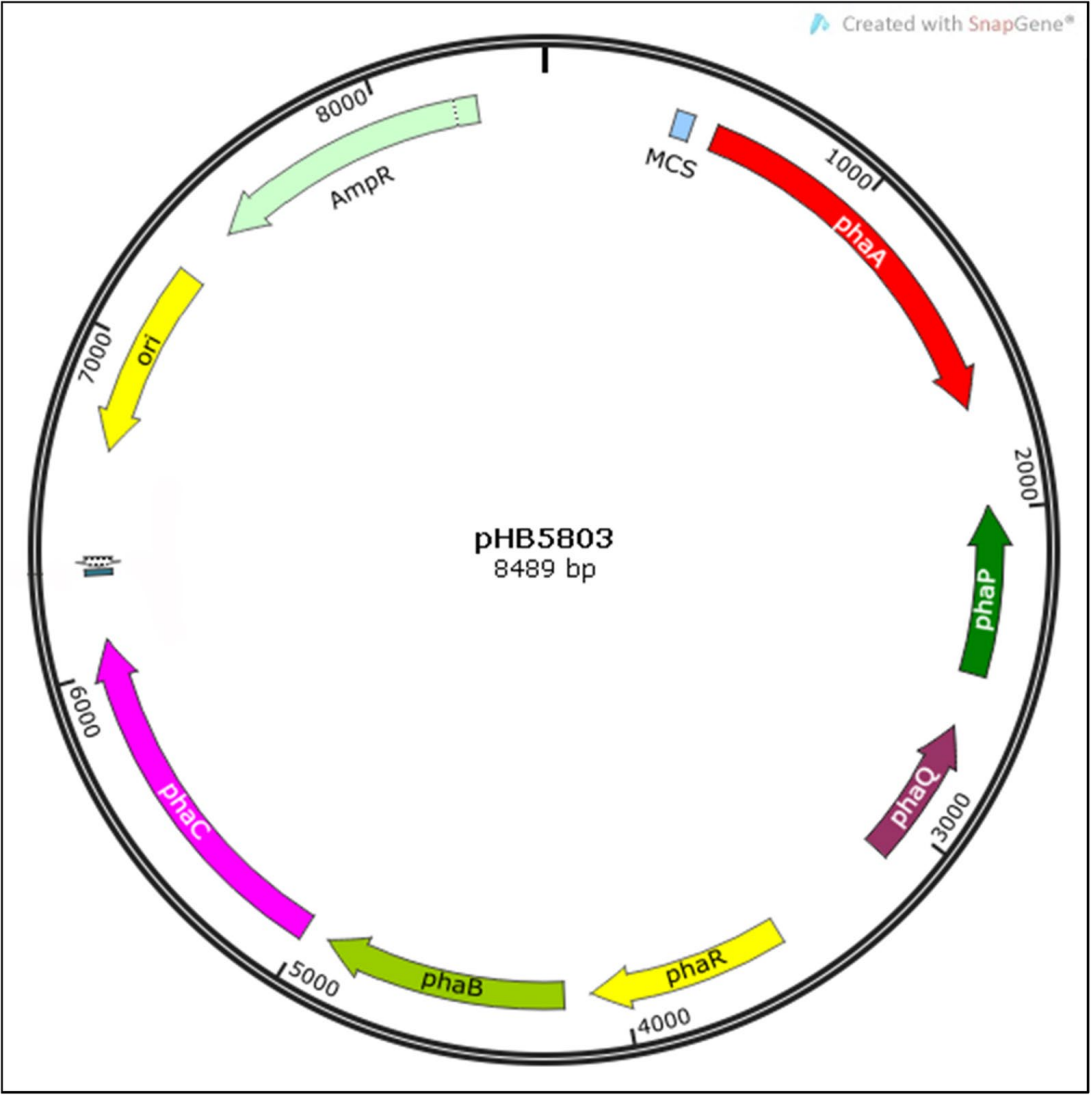
Physical map of plasmid pHB5803 (Generated using SnapGene software, GSL Biotech, Illinois, USA)

**Fig. 2:**
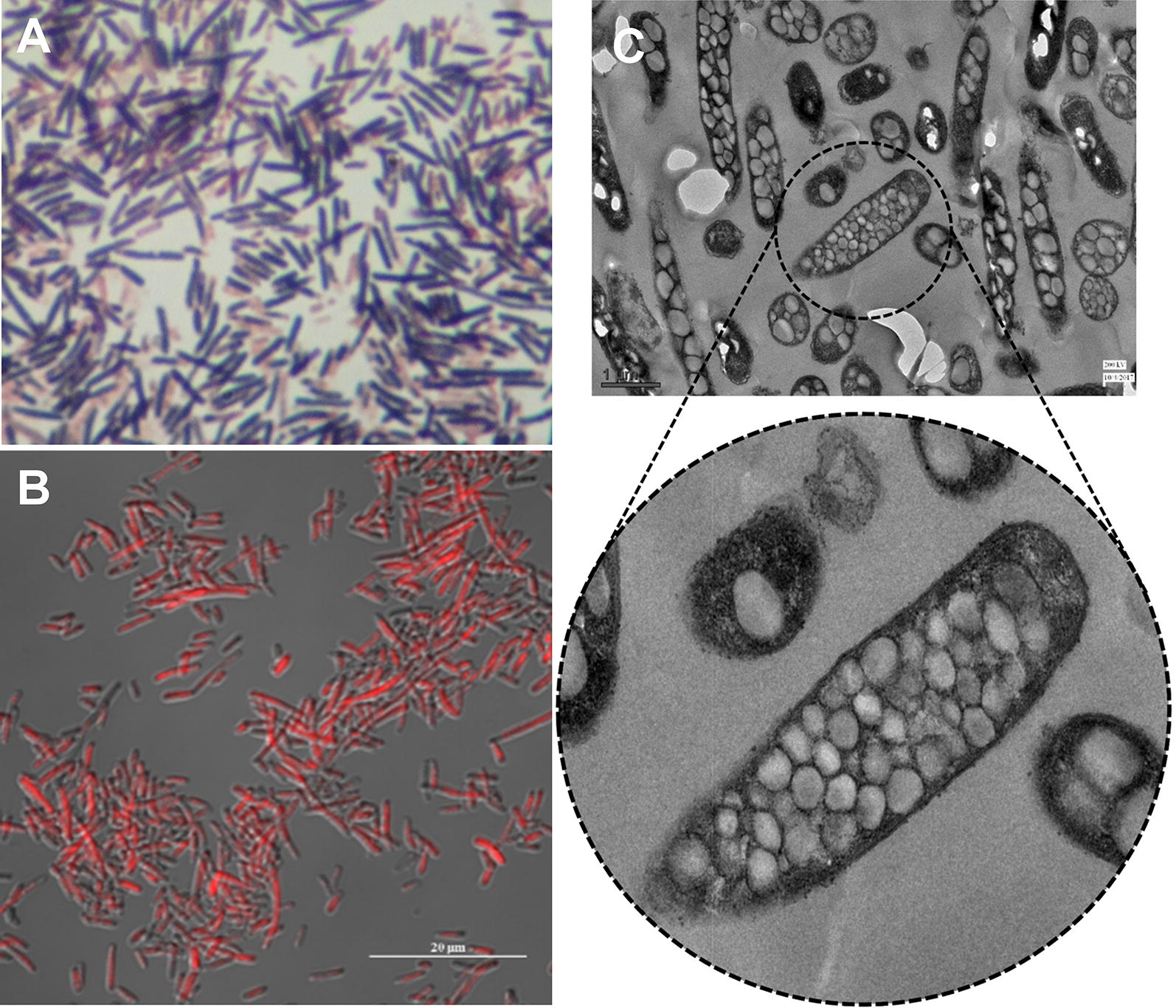
Visualization of polymer accumulation in recombinant *E. coli*. **A** Cells stained with Sudan Black B observed through light microscope. **B** Confocal microscopic images of cells stained with Nile Red (×50 magnification). **C** TEM images of cells with PHB granules (magnified view in the circle, scale bar = 1 μm)

The 4.4 kb long *phaPQRBC* gene cluster and the 1.2 kb *phaA* gene were amplified from *B. aryabhattai* PHB10 with custom designed primer sets. The products were cloned into T/A cloning vectors for DNA sequencing and for convenience in further sub-cloning steps. Both the PHA biosynthesis DNA fragments were inserted into the linearized pUC18 in succession, to get the final recombinant circular vector, pHB5803 (Fig. 1). The recombinant construct was transformed into chemically competent *E. coli* JM109 and the clones were screened for polymer accumulation. Sudan Black B stained culture smear showed cells filled up with darkly stained granules (Fig. 2A) and the cells stained with Nile Red when exposed to UV light, exhibited bright red cytoplasmic granules (Fig. 2B). TEM analysis revealed 10–50 polymer granules having a size range of 0.1–0.3 μm occupying almost the entire recombinant cell volume (Fig. 2C). Polymer accumulation level was estimated as 6.22 ± 0.08 g/L which corresponds to 83.18% of CDM (w/w).

### Characterization of extracted polymer

**Fig. 3:**
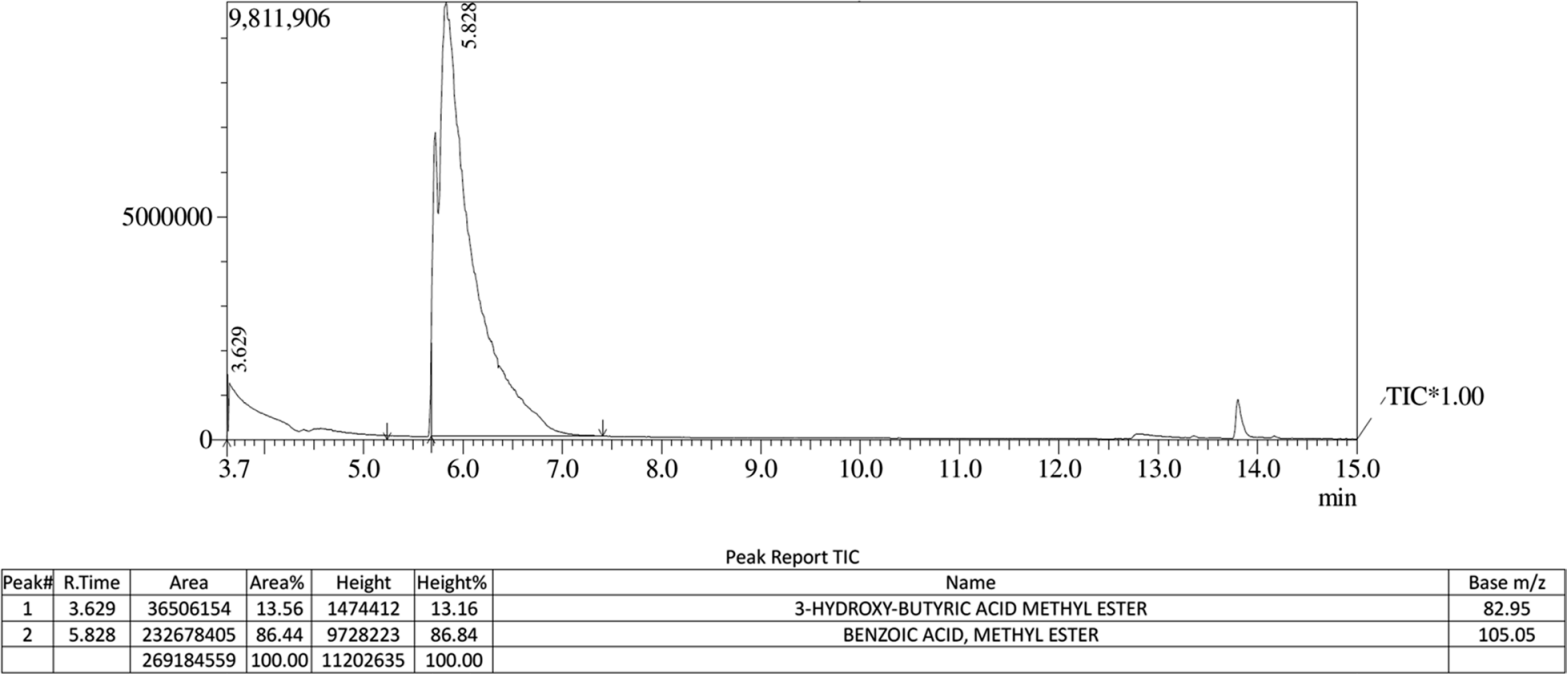
Gas chromatogram of PHB obtained from recombinant *E. coli*

**Fig. 4:**
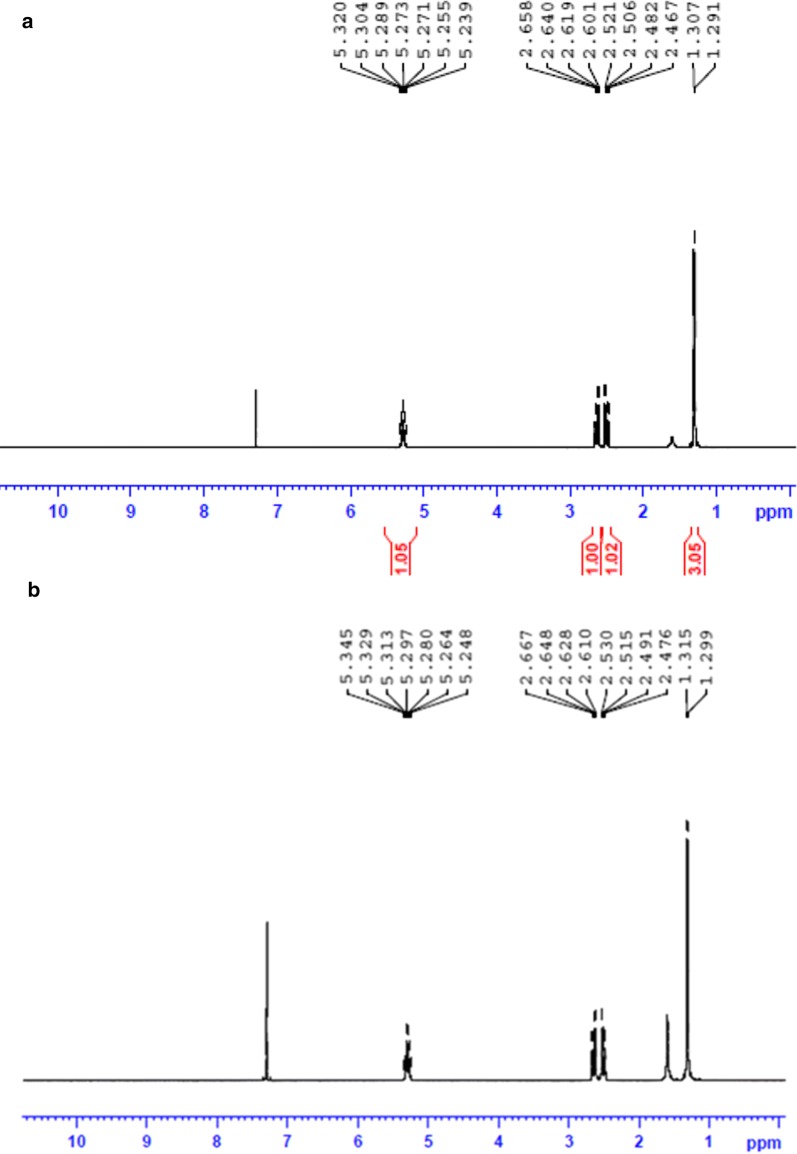
^1^H NMR spectra of the polymer suspended in CDCl_3_. **a** PHB standard (Sigma), **b** PHB from the recombinant strain

**Fig. 5:**
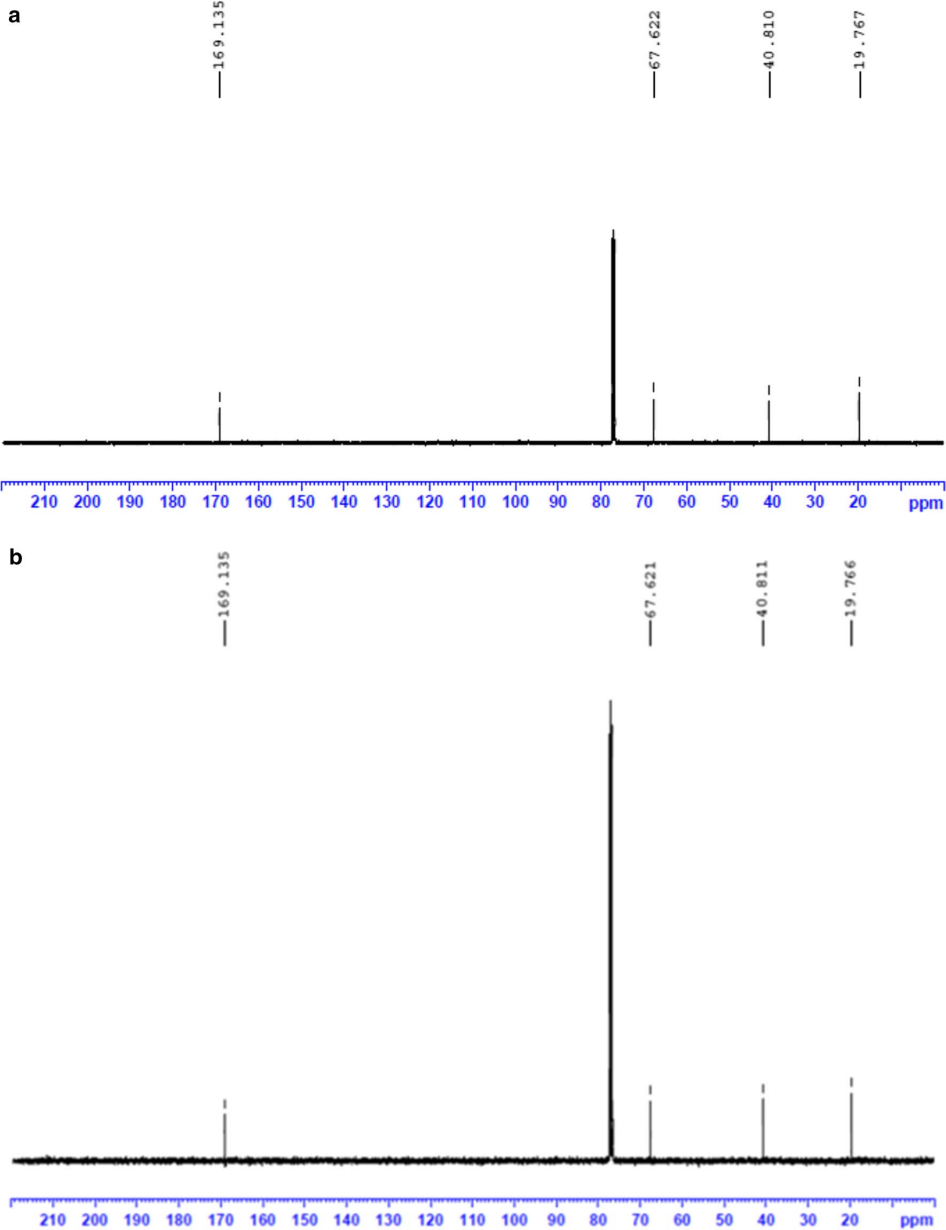
^13^C NMR spectra of the polymer suspended in CDCl_3_. **a** PHB standard (Sigma), **b** PHB from the recombinant strain

**Fig. 6:**
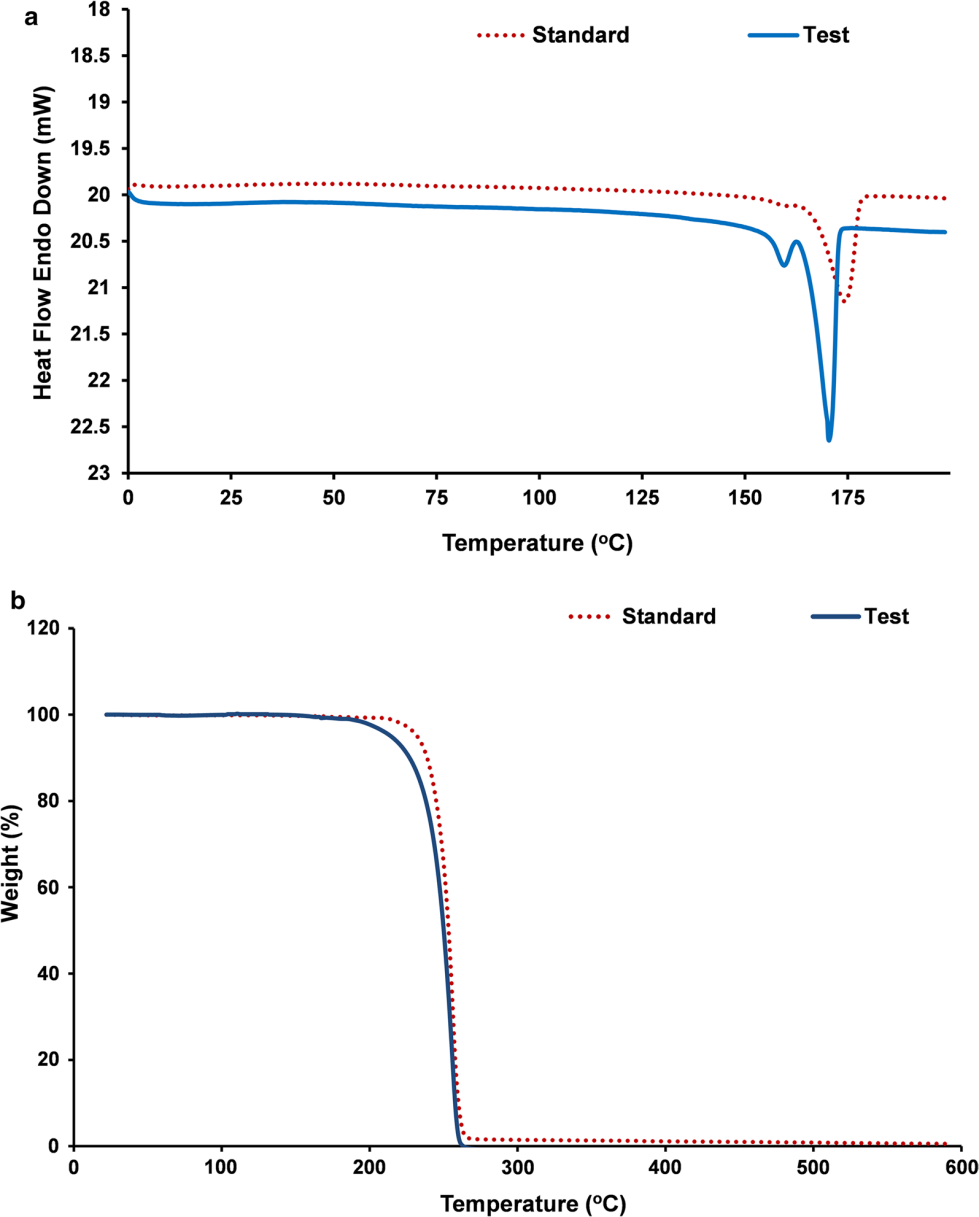
**a** DSC curve of the polymer. **b** TGA curve

Polymer recovered by solvent extraction with chloroform from sodium hypochlorite lysed recombinant *E. coli* cells was characterized. The gas chromatogram of the polymer (Fig. 3) showed a major peak with retention time 3.629 min, which was identified as 3-hydroxy-butyric acid methyl ester by comparing molecules in the GC–MS database. The retention time of internal standard benzoic acid methyl ester was observed as 5.828 min. The ^1^H NMR spectrum (Fig. 4) showed the expected resonances for PHB as demonstrated by a methine group (–CH–) between 5.24 and 5.34 ppm, a methylene group (–CH_2_–) between 2.47 and 2.66 ppm, and a methyl group (–CH_3_) between 1.29 and 1.31 ppm as in standard PHB. Scans of ^13^C NMR (Fig. 5) showed peaks at 169.13, 67.62, 40.81 and 19.76 ppm representing carbonyl carbon (–C–), ester (–O–CH–) group, methylene (–CH_2_–) and methyl (–CH_3_) groups respectively, as in the standard. DSC analysis revealed the melting point of the polymer as 171 °C and its thermal stability at temperature range of 0–140 °C (Fig. 6a). Figure 6b shows the TGA thermogram of the PHA film. The polymer showed no weight loss at temperature up to 200 °C. Thermal degradation occurs after 200 °C and maximum degradation at 260 °C. GPC analysis revealed the number average molecular weight (Mn) of 78.947 kDa and a weight average molecular weight (Mw) of 143.108 kDa with polydispersity index (PDI) 1.81. The tensile strength and elongation at break of the polymer were observed as 14.2 MPa and 7.65% respectively.

### EDTA–microwave assisted cell lysis and polymer extraction

**Fig. 7:**
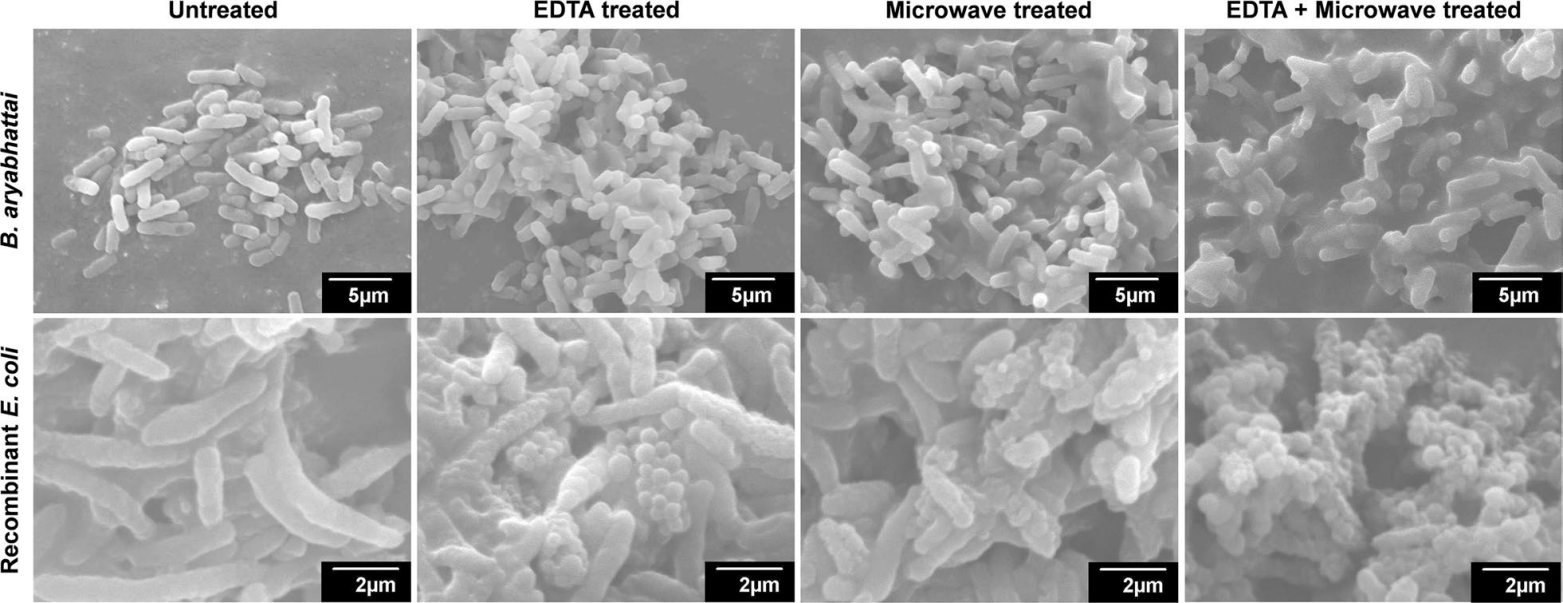
SEM images depicting the effect of EDT and microwave on cell morphology (magnification: *B. aryabhattai*—×3000; recombinant *E. coli*—×5000)

The *B. aryabhattai* PHB10 accumulated PHB at 76.29% of CDM (w/w) in basal medium supplemented with glucose. Biomass obtained from the recombinant *E. coli* as well as the PHB10 cultures was used for the cell lysis experiments. The effect of EDTA and microwave on bacterial cell wall was visualized through SEM (Fig. 7). The images indicated partial disturbances of the *E. coli* cell wall, when the cells were treated with EDTA and subjected to microwave radiation independently. The combined treatment of EDTA and microwave radiation resulted in complete breakage of the cells. But the treatments did not make any significant change in the morphology of the *B. aryabhattai* cells tested. The difference in yield, purity, molecular weight and polydispersity of PHB obtained by solvent extraction after cell disruption by EDTA–microwave and sodium hypochlorite treatments is given in Table 2. The EDTA–microwave method showed a polymer yield of 93.75% with purity of 97.21% from recombinant *E. coli*. The GPC analysis showed 2.9 fold improvement in molecular weight for the PHB extracted from *E. coli*, compared with that of sodium hypochlorite lysed sample. The polydispersity was also enhanced to 1.67 in comparison with 1.81 of the sodium hypochlorite treatment. The molecular weight of polymer from the Gram positive strain *B. aryabhattai* PHB10 was improved by 0.39 fold, but there was an increase in PDI from 2.67 to 3.77.

**Table 2:** Yield, purity, molecular weight and polydispersity of PHB obtained by solvent extraction after cell disruption by EDTA–microwave and sodium hypochlorite treatments

Parameter	*B. aryabhattai*		Recombinant *E. coli*	
	Sodium hypochlorite	EDTA–microwave	Sodium hypochlorite	EDTA–microwave
Yield (%)	89.77 (± 2.04)	51.12 (± 3.12)	96.81 (± 1.47)	93.75 (± 2.61)
Purity (%)	91.22 (± 1.97)	93.01 (± 2.09)	93.64 (± 1.83)	97.21 (± 2.22)
Mw (kDa)	199.740	277.593	143.108	553.366
PDI	2.67	3.77	1.81	1.67

Yield and purity values are means from three independent experiments (standard deviation in parenthesis)

## Discussion

PHA production in recombinant *E. coli* strains has been proved as an ideal strategy for cost effective biopolymer synthesis (Li et al. 2007; Choi et al. 1998). In this study, a recombinant *E. coli* system was developed using PHB biosynthesis genes from *B. aryabhattai* PHB10, which was reported previously as an efficient PHB producer (Pillai et al. 2017a). This wild strain harbours a cluster (*phaPQRBC*) of five genes involved in PHA metabolism viz., *phaP* (polyhydroxyalkanoic acid inclusion protein), *phaQ* (poly-beta-hydroxybutyrate-responsive repressor), *phaR* (polyhydroxyalkanoic acid synthase subunit R), *phaB* (acetoacetyl CoA reductase), and *phaC* [poly(R)-hydroxyalkanoic acid synthase subunit] typical to the genus *Bacillus* (McCool and Cannon 2001; Pillai et al. 2017b). In contrast to the PHB gene cluster in *Ralstonia eutropha*, the gene coding for acetyl-CoA C-acetyltransferase (*phaA*) is not a part of the PHB operon in the genus *Bacillus* and is located elsewhere in the genome. Hence the two DNA fragments *phaPQRBC* and *phaA* were amplified individually and introduced into the plasmid vector, pUC18 for heterologous expression.

The recombinant *E. coli* harbouring pHB5803 accumulated PHA granules in their cytoplasm when cultivated in presence of glycerol and were visualized by the light-fluorescent microscopies and TEM. Similar observations were previously reported in PHA accumulating recombinant *E. coli* (Jari et al. 2015; Bresan et al. 2016; Horng et al. 2011). In the recombinant construct, the PHB genes were inserted along with their own original promoter sequences from the wild strain and hence the genes were expressed without external supply of IPTG for induction. At large scale PHA production process, recombinant systems expressing without IPTG induction will be helpful in reducing the polymer production cost (Lee and Keasling 2006).

Genetic elements from Gram negative bacteria are generally of high preference while attempting heterologous production of PHAs in *E. coli*, as they are more compatible with in *E. coli* protein expression machinery (Agnew et al. 2012; Le Meur et al. 2013). Hence there have been only a limited number of reports on the construction of recombinant vector system for PHB production in *E. coli* using PHB biosynthesis genes from Gram positive bacteria. Nevertheless, we have achieved a maximum polymer accumulation level 6.22 ± 0.08 g/L (83.18% of CDM) in the recombinant strain engineered solely with the genes taken from a *Bacillus* sp. Mahishi et al. (2003) have constructed a recombinant *E. coli* system with *Streptomyces aureofaciens*, and demonstrated the accumulation of PHB using genes from a Gram positive bacterium with polymer yield of 60% of CDM. Later, Desetty et al. (2008) constructed a recombinant plasmid using the PHB gene cluster from *Bacillus thuringiensis* and reported 24% PHB accumulation in *E. coli*. However, they have complemented the locus with *phaA* from *R. eutropha* to achieve this polymer production level. Davis et al. (2008) have attempted to clone *Pseudomonas aeruginosa* genes PHA synthase1 (*phaC1*) and (R)-specific enoyl CoA hydratase1 (*phaJ1*) along with *phaA* and *phaB* from a *Bacillus* sp. The cloning resulted in accumulation of PHB and mcl-PHA in *E. coli*. In another study, Tomizawa et al. (2011) have engineered a recombinant construct harbouring *phaR* and *phaC* from *Bacillus cereus* complemented with *phaA* and *phaB* from *R. eutropha* to yield 7.44 g/L of PHB. Recently, El Rabey et al. (2017) have reported the construction of a recombinant system exclusively using the PHB synthesis genetic background from *B. cereus*, but PHB accumulation was not mentioned. A few other studies on PHB production by recombinant *E. coli* incorporating genetic elements from Gram negative bacteria have reported a maximum yield of 0.21 g/L and 2.02 g/L (Arifin et al. 2011; de Almeida et al. 2010). The novelty of the recombinant *E. coli* strain harbouring pHB5803 discussed in this study is the highest level of PHB accumulation reported till date, solely expressing the genes from a Gram positive bacterium. The value was also found to be higher when compared to the yield (3.26 g/L) reported from the wild strain *B. aryabhattai* PHB10 (Pillai et al. 2017a).

The plasmid pUC18 was used as the vector for the construction of pHB5803 and requires ampicillin as selection pressure to maintain stability of the recombinant plasmid. Antibiotics supplementation during fermentation is an important factor contributing to the overall polymer production cost, which is not desirable at industrial levels of PHB production. Integration of PHB biosynthesis operon into *E. coli* chromosome by homologous recombination is an established strategy (Lee and Lee 2003) to overcome this problem. For an industrial production process, the recombinant strain used should be capable of accumulating very high polymer content in their cells than the normal laboratory strains. Genetically engineered *E. coli*, with some deletions such as *mtgA* and *mreB* were observed to be improving PHB production via enhanced inclusion body accumulation and cell enlargement (Jiang et al. 2015; Kadoya et al. 2015). Integration of amylase gene into recombinant *E. coli* is a strategy to make use of starch as the sole carbon source for PHB production (Bhatia et al. 2015). Application of these techniques in the recombinant strain developed in this study may improve it’s the industrial applicability.

GC–MS analysis revealed the monomer composition of the extracted polymer. The peak obtained at 3.629 min represented butyric acid methyl ester indicating the material is a homopolymer of 3-hydroxy-butyric acid, i.e., poly-3-hydroxybutyrate. The ^1^H NMR spectrum demonstrated the resonances for the methine (–CH–), methylene (–CH_2_–) and methyl (–CH_3_) groups of poly(3-hydroxybutyrate) as obtained in the standard PHB sample. The resonances for the methylene (–CH_2_–), methyl (–CH_3_), ester (–O–CH–) groups and the carbonyl carbon (–C–) atom were demonstrated by ^13^C NMR. The analyses proved the polymer from the recombinant bacterial culture was PHB and the resonance values were in agreement with the observations of Salgaonkar et al. (2013). The DSC and TGA analyses revealed the thermal properties of the extracted polymer as stable in a temperature range of 0–171 °C and resistant to mass loss upon heating up to 200 °C. These observations proved that the polymer from the recombinant *E. coli* is of good thermal characteristics matching with commercial grade PHB. However the molecular weight and PDI of the extracted polymer were found to be inferior when compared to previous reports on PHB production in recombinant *E. coli* (Nikel et al. 2006; Phithakrotchanakoon et al. 2013). The sodium hypochlorite mediated extraction would have affected the polymer chain length and was reflected in the lower molecular weight and PDI (Hahn et al. 1994). The polymer has a tensile strength of 14.2 MPa and an elongation at break of 7.65%. Tensile strength is the stress required to break a sample and the elongation at break is the strain when it ruptures. The values obtained is found lower when compared to the standard PHB, which were calculated to be 28 MPa and 9% respectively (Parra et al. 2006). This lower mechanical strength may be due to the low molecular weight of the extracted polymer, as the mechanical strength of PHB is positively correlated with its molecular weight (Iwata 2005).

Sodium hypochlorite is a highly preferred cell lysing agent in the recovery of PHAs from harvested cell mass (Heinrich et al. 2012). Despite of its several advantages, it severely affects the molecular weight and PDI of the extracted polymer which deteriorates the processibility of the polymer. This demands new cell lysis methods for better polymer recovery. The impact of EDTA and microwave radiation on bacterial cell wall has been reported previously (Gray and Wilkinson 1965; Woo et al. 2000) and their effect on PHA extraction from bacterial cells was tested during this study on the recombinant as well as the wild strain. The SEM observation suggested that an integrated application of EDTA and microwave radiation can disintegrate *E. coli* cells aiding the release of accumulated polymer granules from the cytoplasm. Previous reports stated that the damage caused on the *E. coli* cells are due to the specific electromagnetic effects of microwave radiation rather than its thermal effect (Shamis et al. 2011). The same treatment was found insufficient to lyse the Gram positive *B. aryabhattai* cells for polymer recovery.

The purity of PHB (97.21%) attained by the EDTA–microwave mediated lysis was comparable to the value (98.3%) obtained through chloroform extraction by Ibrahim and Steinbüchel (2009) and is higher than the value (95.66%) obtained through sodium hypochlorite extraction as reported by Heinrich et al. (2012). The polymer yield by EDTA–microwave method (93.75%) is better than the previously reported values 85.0% and 91.32% (Ibrahim and Steinbüchel 2009; Heinrich et al. 2012). However the yield was a little lower in comparison to the hypochlorite mediated extraction done as the control experiment, which may be due to the polymer loss at the filtration step, but the purity was better. The *B. aryabhattai* cells were resistant to the lysis method which reflected in the polymer yield. This might be due to the thick cell wall content in the Gram positive bacterium which resisted the combined EDTA–microwave treatment. The pioneer studies in this field (Gray and Wilkinson 1965; Woo et al. 2000) also reported the ineffectiveness of EDTA and microwave radiation in making damage to Gram positive cell walls. Recently, Akdoğan and Çelik (2018) reported the effect of microwaving as a method of dehydration for biomass pre-treatment during solvent extraction of PHA. They concluded that the method is more effective and economical than other drying methods such as freeze-drying and ethanol/heat-treatment.

The polymer extracted from the recombinant strain by the combined EDTA–microwave method showed a 2.9-fold improvement in molecular weight (Mw) with lower PDI, in comparison to the conventional hypochlorite lysis method. PDI is a measure of homogeneity of a polymer, which indicates that the extracted polymer chains were more intact than from the conventional method. Sodium hypochlorite causes severe degradation of polymer chains during cell lysis (Hahn et al. 1994) which might be the reason for the reduction in molecular weight of the polymer obtained through this method. The EDTA–microwave assisted lysis did not use sodium hypochlorite and thereby improved the molecular weight and PDI of the recovered polymer.

EDTA is considered as an environmental pollutant, if it is released into the surroundings (Oviedo and Rodríguez 2003), but it is not acutely toxic to aquatic organisms, known to be biodegradable under certain conditions and the EDTA-Fe(III) chelates in the environment are also susceptible to photo-degradation (Wolf and Gilbert 1992; Sillanpää 1997). After the separation of the polymer and cell debris, the EDTA solution can be reused one or two times which will considerably reduce the overall quantity released into the environment. Hence the EDTA–microwave assisted extraction method described here can be considered environmental friendly. However, the process still utilized chloroform at the final stage of the recovery process which needs to be avoided to make the process applicable to industrial levels in a more environment friendly manner. The present study is the first attempt to evaluate the efficacy of EDTA and microwaves in release of PHAs from bacterial cells.

PHB accumulating recombinant *E. coli* strain was engineered by expressing *B. aryabhattai* PHB10 PHA-biosynthesis gene cluster. The strain did not need external induction for PHB production. The polymer accumulation was visualized by different microscopic techniques and the polymer yield is the highest known value for a recombinant system employing solely *Bacillus* PHA biosynthesis genes. The physical as well as thermal properties of the polymer were studied and found as of good quality, comparable to that of commercial grade PHB. An alternative EDTA–microwave assisted cell lysis method was evaluated for polymer recovery from recombinant *E. coli* cells. The PHB extracted through this approach was of higher molecular weight, better PDI and purity when compared to sodium hypochlorite lysis mediated extraction.

## Abbreviations

PHB: poly(3-hydroxybutyrate)
PHA: polyhydroxyalkanoate
NMR: nuclear magnetic resonance
PDI: polydispersity index
EDTA: ethylenediaminetetraacetic acid
IPTG: isopropyl-β-d-thiogalactopyranoside
CDM: cell dry mass
SEM: scanning electron microscopy
TEM: transmission electron microscopy
GC–MS: gas chromatography–mass spectrometry
DSC: differential scanning calorimetry
TGA: thermo gravimetric analysis
GPC: gel permeation chromatography

## References

[CR1] Agnew DE, Stevermer AK, Youngquist JT, Pfleger BF (2012). Engineering *Escherichia coli* for production of C_12_–C_14_ polyhydroxyalkanoate from glucose. Metab Eng.

[CR2] Akai M, Onai K, Kusano M, Sato M, Redestig H, Toyooka K, Morishita M, Miyake H, Hazama A, Checchetto V, Szabò I, Matsuoka K, Saito K, Yasui M, Ishiura M, Uozumi N (2011). Plasma membrane aquaporin AqpZ protein is essential for glucose metabolism during photomixotrophic growth of *Synechocystis* sp. PCC 6803. J Biol Chem.

[CR3] Akdoğan M, Çelik E (2018). Purification and characterization of polyhydroxyalkanoate (PHA) from *Bacillus megaterium* strain using various dehydration techniques. J Chem Technol Biotechnol.

[CR4] Aneesh BP, Arjun JK, Kavitha T, Harikrishnan K (2016). Production of short chain length polyhydroxyalkanoates by *Bacillus megaterium* PHB29 from starch feed stock. Int J Curr Microbiol App Sci.

[CR5] Arifin Y, Sabri S, Sugiarto H, Krömer JO, Vickers CE, Nielsen LK (2011). Deletion of cscR in *Escherichia coli* W improves growth and poly-3-hydroxybutyrate (PHB) production from sucrose in fed batch culture. J Biotechnol.

[CR6] Berger E, Ramsay BA, Ramsay JA, Chavarie C, Braunegg G (1989). PHB recovery by hypochlorite digestion of non-PHB biomass. Biotechnol Tech.

[CR7] Bhatia SK, Shim YH, Jeon JM, Brigham CJ, Kim YH, Kim HJ, Seo HM, Lee JH, Kim JH, Yi DH, Lee YK (2015). Starch based polyhydroxybutyrate production in engineered *Escherichia coli*. Bioprocess Biosyst Eng.

[CR8] Bhattacharyya A, Pramanik A, Maji SK, Haldar S, Mukhopadhyay UK, Mukherjee J (2012). Utilization of vinasse for production of poly-3-(hydroxybutyrate-*co*-hydroxyvalerate) by *Haloferax mediterranei*. AMB express.

[CR9] Bresan S, Sznajder A, Hauf W, Forchhammer K, Pfeiffer D, Jendrossek D (2016). Polyhydroxyalkanoate (PHA) granules have no phospholipids. Sci Rep.

[CR10] Burdon KL (1946). Fatty material in bacteria and fungi revealed by staining dried, fixed slide preparations. J Bacteriol.

[CR11] Choi JI, Lee SY (1999). Efficient and economical recovery of poly (3-hydroxybutyrate) from recombinant *Escherichia coli* by simple digestion with chemicals. Biotechnol Bioeng.

[CR12] Choi JI, Lee SY, Han K (1998). Cloning of the *Alcaligenes latus* polyhydroxyalkanoate biosynthesis genes and use of these genes for enhanced production of poly (3-hydroxybutyrate) in *Escherichia coli*. Appl Environ Microbiol.

[CR13] Davis R, Anilkumar PK, Chandrashekar A, Shamala TR (2008). Biosynthesis of polyhydroxyalkanoates co-polymer in *E. coli* using genes from *Pseudomonas* and *Bacillus*. Antonie Van Leeuwenhoek.

[CR14] de Almeida A, Giordano AM, Nikel PI, Pettinari MJ (2010). Effects of aeration on the synthesis of poly (3-hydroxybutyrate) from glycerol and glucose in recombinant *Escherichia coli*. Appl Environ Microbiol.

[CR15] Desetty RD, Mahajan VS, Khan BM, Rawal SK (2008). Isolation and heterologous expression of PHA synthesising genes from *Bacillus thuringiensis* R1. World J Microbiol Biotechnol.

[CR16] El Rabey HA, Albureikan MO, Aly MM, Kabli SA, Schneider K, Nolke G (2017). Isolation, cloning and sequencing of poly 3-hydroxybutyrate synthesis genes from local strain of *Bacillus cereus* mm7 and expressing them in *E. coli*. J Investig Genomics.

[CR17] Fiorese ML, Freitas F, Pais J, Ramos AM, de Aragão GM, Reis MA (2009). Recovery of polyhydroxybutyrate (PHB) from *Cupriavidus necator* biomass by solvent extraction with 1,2-propylene carbonate. Eng Life Sci.

[CR18] Gao D, Maehara A, Yamane T, Ueda S (2001). Identification of the intracellular polyhydroxyalkanoate depolymerase gene of *Paracoccus denitrificans* and some properties of the gene product. FEMS Microbiol Lett.

[CR19] Gray GW, Wilkinson SG (1965). The effect of ethylenediaminetetra-acetic acid on the cell walls of some Gram-negative bacteria. Microbiology.

[CR20] Griffin G (1994). Chemistry and technology of biodegradable polymers.

[CR21] Gunaratne LMWK, Shanks RA, Amarasinghe G (2004). Thermal history effects on crystallisation and melting of poly (3-hydroxybutyrate). Thermochim Acta.

[CR22] Hahn SK, Chang YK, Kim BS, Chang HN (1994). Optimization of microbial poly (3-hydroxybutyrate) recover using dispersions of sodium hypochlorite solution and chloroform. Biotechnol Bioeng.

[CR23] Hanahan D, Glover DM (1985). Techniques for transformation of *Escherichia coli*. DNA cloning: a practical approach.

[CR24] Heinrich D, Madkour MH, Al-Ghamdi MA, Shabbaj II, Steinbüchel A (2012). Large scale extraction of poly (3-hydroxybutyrate) from *Ralstonia eutropha* H16 using sodium hypochlorite. AMB Express.

[CR25] Horng YT, Chien CC, Wei YH, Chen SY, Lan JW, Sun YM, Soo PC (2011). Functional cis-expression of phaCAB genes for poly (3-hydroxybutyrate) production by *Escherichia coli*. Lett Appl Microbiol.

[CR26] Ibrahim MH, Steinbüchel A (2009). Poly (3-hydroxybutyrate) production from glycerol by *Zobellella denitrificans* MW1 via high-cell-density fed-batch fermentation and simplified solvent extraction. Appl Environ Microbiol.

[CR27] Iwata T (2005). Strong fibers and films of microbial polyesters. Macromol Biosci.

[CR28] Jari M, Khatami SR, Galehdari H, Shafiei M (2015). Cloning and expression of poly 3-hydroxybutyrate operon into *Escherichia coli*. Jundishapur J Microbiol.

[CR29] Jendrossek D, Selchow O, Hoppert M (2007). Poly(3-hydroxybutyrate) granules at the early stages of formation are localized close to the cytoplasmic membrane in *Caryophanon latum*. Appl Environ Microbiol.

[CR30] Jiang XR, Wang H, Shen R, Chen GQ (2015). Engineering the bacterial shapes for enhanced inclusion bodies accumulation. Metab Eng.

[CR31] Juengert JR, Bresan S, Jendrossek D (2018). Determination of polyhydroxybutyrate (PHB) content in *Ralstonia eutropha* using gas chromatography and Nile Red staining. Bio-protocol.

[CR32] Kadoya R, Matsumoto KI, Ooi T, Taguchi S (2015). MtgA deletion-triggered cell enlargement of *Escherichia coli* for enhanced intracellular polyester accumulation. PLoS ONE.

[CR33] Kapritchkoff FM, Viotti AP, Alli RCP, Zuccolo M, Pradella JGC, Maiorano AE, Miranda EA, Bonomi A (2006). Enzymatic recovery and purification of polyhydroxybutyrate produced by *Ralstonia eutropha*. J Biotechnol.

[CR34] Khanna S, Srivastava AK (2005). Recent advances in microbial polyhydroxyalkanoates. Process Biochem.

[CR35] Law JH, Slepecky RA (1961). Assay of poly-β-hydroxybutyric acid. J Bacteriol.

[CR36] Le Meur S, Zinn M, Egli T, Thöny-Meyer L, Ren Q (2013). Poly(4-hydroxybutyrate) (P4HB) production in recombinant *Escherichia coli*: P4HB synthesis is uncoupled with cell growth. Microb Cell Fact.

[CR37] Lee SY (1996). High cell-density culture of *Escherichia coli*. Trends Biotechnol.

[CR38] Lee SY (1997). *E. coli* moves into the plastic age. Nature.

[CR39] Lee SY, Chang HN, Fiechter A (1995). Production of poly(hydroxyalkanoic acid). Microbial and eznymatic bioproducts.

[CR40] Lee SK, Keasling JD (2006). Propionate-regulated high-yield protein production in *Escherichia coli*. Biotechnol Bioeng.

[CR41] Lee SY, Lee Y (2003). Metabolic engineering of *Escherichia coli* for production of enantiomerically pure (R)-(−)-hydroxycarboxylic acids. Appl Environ Microbiol.

[CR42] Lee SY, Lee KM, Chan HN, Steinbüchel A (1994). Comparison of recombinant *Escherichia coli* strains for synthesis and accumulation of poly-(3-hydroxybutyric acid) and morphological changes. Biotechnol Bioeng.

[CR43] Li R, Zhang H, Qi Q (2007). The production of polyhydroxyalkanoates in recombinant *Escherichia coli*. Bioresour Technol.

[CR44] Madison LL, Huisman GW (1999). Metabolic engineering of poly(3-hydroxyalkanoates): from DNA to plastic. Microbiol Mol Biol Rev.

[CR45] Mahishi LH, Tripathi G, Rawal SK (2003). Poly(3-hydroxybutyrate) (PHB) synthesis by recombinant *Escherichia coli* harbouring *Streptomyces aureofaciens* PHB biosynthesis genes: effect of various carbon and nitrogen sources. Microbiol Res.

[CR46] McCool GJ, Cannon MC (2001). PhaC and PhaR are required for polyhydroxyalkanoic acid synthase activity in *Bacillus megaterium*. J Bacteriol.

[CR47] Muhammadi Shabina, Afzal M, Hameed S (2015). Bacterial polyhydroxyalkanoates-eco-friendly next generation plastic: production, biocompatibility, biodegradation, physical properties and applications. Green Chem Lett Rev.

[CR48] Nikel PI, de Almeida A, Melillo EC, Galvagno MA, Pettinari MJ (2006). New recombinant *Escherichia coli* strain tailored for the production of poly (3-hydroxybutyrate) from agroindustrial by-products. Appl Environ Microbiol.

[CR49] Oviedo C, Rodríguez J (2003). EDTA: the chelating agent under environmental scrutiny. Quím Nova.

[CR50] Parra DF, Fusaro J, Gaboardi F, Rosa DS (2006). Influence of poly (ethylene glycol) on the thermal, mechanical, morphological, physical–chemical and biodegradation properties of poly (3-hydroxybutyrate). Polym Degrad Stabil.

[CR51] Phithakrotchanakoon C, Champreda V, Aiba S, Pootanakit K, Tanapongpipat S (2013). Engineered *Escherichia coli* for short-chain-length medium-chain-length polyhydroxyalkanoate copolymer biosynthesis from glycerol and dodecanoate. Biosci Biotechnol Biochem.

[CR52] Pillai AB, Kumarapillai HK, Shukla P (2017). Bacterial polyhydroxyalkanoates: recent trends in production and applications. Recent advances in applied microbiology.

[CR53] Pillai AB, Kumar AJ, Thulasi K, Kumarapillai H (2017). Evaluation of short-chain-length polyhydroxyalkanoate accumulation in *Bacillus aryabhattai*. Braz J Microbiol.

[CR54] Pillai AB, Kumar AJ, Thulasi K, Reghunathan D, Prasannakumar M, Kumarapillai H (2017). Draft genome sequence of *Bacillus aryabhattai* strain PHB10, a poly(3-hydroxybutyrate)-accumulating bacterium isolated from domestic sewerage. Genome Announc.

[CR55] Pötter M, Steinbüchel A (2005). Poly (3-hydroxybutyrate) granule-associated proteins: impacts on poly (3-hydroxybutyrate) synthesis and degradation. Biomacromolecules.

[CR56] Qi Q, Rehm BH (2001). Polyhydroxybutyrate biosynthesis in *Caulobacter crescentus*: molecular characterization of the polyhydroxybutyrate synthase. Microbiology.

[CR57] Ramsay JA, Berger E, Voyer R, Chavarie C, Ramsay BA (1994). Extraction of poly-3-hydroxybutyrate using chlorinated solvents. Biotechnol Tech.

[CR58] Rehm BH (2003). Polyester synthases: natural catalysts for plastics. Biochem J.

[CR59] Salgaonkar BB, Mani K, Braganca JM (2013). Characterization of polyhydroxyalkanoates accumulated by a moderately halophilic salt pan isolate *Bacillus megaterium* strain H16. J Appl Microbiol.

[CR60] Sambrook J, Russell DW (2001). Molecular cloning: a laboratory manual.

[CR61] Samori C, Abbondanzi F, Galletti P, Giorgini L, Mazzocchetti L, Torri C, Tagliavini E (2015). Extraction of polyhydroxyalkanoates from mixed microbial cultures: impact on polymer quality and recovery. Bioresour Technol.

[CR62] Samori C, Basaglia M, Casella S, Favaro L, Galletti P, Giorgini L, Marchi D, Mazzocchetti L, Tagliavini C, Torri E (2015). Dimethyl carbonate and switchable anionic surfactants: two effective tools for the extraction of polyhydroxyalkanoates from microbial biomass. Green Chem.

[CR63] Shamis Y, Taube A, Mitik-Dineva N, Croft R, Crawford RJ, Ivanova EP (2011). Specific electromagnetic effects of microwave radiation on *Escherichia coli*. Appl Environ Microbiol.

[CR64] Sharma R, Ray AR (1995). Polyhydroxybutyrate, its copolymers and blends. J Macromol Sci Polymer Rev.

[CR65] Shi H, Shiraishi M, Shimizu K (1997). Metabolic flux analysis for biosynthesis of poly(-hydroxybutyric acid) in *Alcaligenes eutrophus* from various carbon sources. J Ferment Bioeng.

[CR66] Sillanpää M, Ware GW (1997). Environmental fate of EDTA and DTPA. Reviews of environmental contamination and toxicology.

[CR67] Soo-Hwan K, Lee HS, Ryu DS, Choi SJ, Lee DS (2011). Antibacterial activity of silver-nanoparticles against *Staphylococcus aureus* and *Escherichia coli*. Korean J Microbiol Biotechnol.

[CR68] Su WF (2013) Polymer size and polymer solutions. In: Principles of polymer design and synthesis. Lecture Notes in Chemistry. Springer, Berlin, pp 9–26. 10.1007/978-3-642-38730-2_2

[CR69] Tamer IM, Moo-Young M, Chisti Y (1998). Disruption of *Alcaligenes latus* for recovery of poly(β-hydroxybutyric acid): comparison of high-pressure homogenization, bead milling, and chemically induced lysis. Ind Eng Chem Res.

[CR70] Tomizawa S, Hyakutake M, Saito Y, Agus J, Mizuno K, Abe H, Tsuge T (2011). Molecular weight change of polyhydroxyalkanoate (PHA) caused by the PhaC subunit of PHA synthase from *Bacillus cereus* YB-4 in recombinant *Escherichia coli*. Biomacromolecules.

[CR71] Urtuvia V, Villegas P, González M, Seeger M (2014). Bacterial production of the biodegradable plastics polyhydroxyalkanoates. Int J Biol Macromol.

[CR72] Wolf K, Gilbert PA, de Oude NT (1992). EDTA-ethylenediaminetetraacetic acid. Detergents. Anthropogenic compounds.

[CR73] Woo IS, Rhee IK, Park HD (2000). Differential damage in bacterial cells by microwave radiation on the basis of cell wall structure. Appl Environ Microbiol.

